# Surface Modification of Polyetheretherketone (PEEK) via Femtosecond Laser Microprocessing for Enhanced Bioactivity: A Preliminary Study

**DOI:** 10.3390/bioengineering12121285

**Published:** 2025-11-23

**Authors:** Liliya Angelova, Emil Filipov, Georgi Avdeev, Albena Daskalova

**Affiliations:** 1 Institute of Electronics, Bulgarian Academy of Sciences, 1784 Sofia, Bulgaria; emil.filipov95@gmail.com (E.F.); albdaskalova@gmail.com (A.D.); 2 Institute of Physical Chemistry, Bulgarian Academy of Sciences, 1113 Sofia, Bulgaria; g_avdeev@ipc.bas.bg

**Keywords:** polyetheretherketone (PEEK), femtosecond (fs) laser hierarchical structuring, surface biofunctionalization, bioactivity, osseointegration

## Abstract

The increasing prevalence of orthopedic disorders and technological advances have significantly improved the design and functionality of orthopedic implants, fostering the growth of the orthopedic implant market. Polyetheretherketone (PEEK) has emerged as a promising alternative to the gold standard of metallic implants due to its favorable biocompatibility and mechanical properties, comparable to those of bone tissue. However, its chemical inertness results in poor osseointegration. This study investigates femtosecond (fs) laser-induced micro- and nanoscale surface modifications of PEEK, aiming to develop surface modifications potentially favorable for bioactivity enhancement of the as-created transient cellular scaffolds. Various texturing designs were fabricated by precisely controlling the laser parameters applied (laser beam power P = 20–80 mW, hatch spacing dx = 45–100 µm, scanning velocity V = 3.44–32 mm/s). The resulting morphologies were characterized by SEM, EDX, XRD, micro-Raman, 3D profilometry, water contact angle measurements, and evaluated for preliminary biological response. The main achievement of the research indicates that the hierarchical topography created by fs laser microprocessing significantly increased surface morphology, which may subsequently provide surface conditions supporting successful osseointegration. These findings demonstrate the feasibility of femtosecond laser structuring as a promising, reproducible, and environmentally friendly method for sustainable surface biofunctionalization of PEEK in orthopedic applications.

## 1. Introduction

In recent decades, the increasing prevalence of orthopedic disorders, combined with advances in materials science and manufacturing technologies, has significantly enhanced the design and functionality of bone implants [1,2]. Among the materials used—metals, ceramics, polymers, and composites—polymers from the polyaryletherketone family, and, in particular, polyetheretherketone itself (PEEK), have gained a prominent position due to their high biocompatibility, excellent mechanical characteristics, and elastic modulus and density close to that of natural bone tissue [3,4,5]. Owing to these properties, PEEK has emerged as a key alternative to metallic implants, which are still considered the “gold standard” in bone tissue engineering [6]. However, the chemical inertness of PEEK represents a major limitation, resulting in poor osseointegration and insufficient long-term stability of the implant within the host bone tissue [7,8]. Due to the absence of bioactive surface groups, targeted surface modification is required to improve biocompatibility, which is a key factor for improved bioactivity and cell adhesion [9].

In recent years, the rapid development of micro- and nanostructuring methods using ultrashort laser pulses has opened new possibilities for fine control of surface morphology, roughness, and hydrophilic-hydrophobic balance in polymer-based biomaterials [10,11,12]. Femtosecond laser processing is distinguished by its unique combination of high spatial resolution, minimal thermal damage, and the ability to reproducibly generate micro- and nanoscale topographies with a permanent effect on the surface morphology [13]. The interplay between wettability and surface roughness is the basis for achieving enhanced osteointegration of implants. In this context, the femtosecond laser processing method represents a unique tool for producing micro- and nano-rough surfaces with improved interface adhesion characteristics [14,15]. The high precision and tunability of the laser radiation parameters provide a possibility for creating diverse micro- and nano-scale surface designs, resembling an effective and environmentally friendly alternative to existing chemical methods by offering permanent surface structuring without residual toxicity. By precisely tuning laser parameters (wavelength, pulse energy, repetition rate, and scanning speed), various textures can be created that may enhance cell adhesion, could stimulate osteogenesis, and provide optimal surface conditions for implant integration [14,15]. Due to the ultra-short laser pulse durations and concentrated high intensities, the created structures offer several advantages, including precise, clean ablation, which ensures control over surface roughness and wettability, high processing accuracy, and reproducibility.

Compared to conventional surface modification methods such as plasma activation, chemical hydroxylation, or sandblasting/acid-etching, femtosecond laser texturing offers a unique combination of precision, reproducibility, and long-term stability [8,16]. Traditional plasma or chemical activation processes can temporarily increase surface energy and introduce hydrophilic functional groups, yet these effects often diminish over time due to surface reorganization or contamination and may leave residual species that compromise biocompatibility [17]. Similarly, abrasive or acid-etching techniques can improve roughness but tend to produce irregular topographies and may induce microcracks or alter the mechanical properties of PEEK, particularly under aggressive conditions [18]. In contrast, femtosecond laser treatment achieves stable hierarchical micro/nanostructures with minimal thermal degradation and without chemical residues, enabling consistent control of wettability and protein adsorption over extended periods [8]. Furthermore, the non-contact and maskless nature of the fs-laser approach supports its scalability and integration into precision manufacturing workflows, making it a reliable alternative to conventional surface treatments for biocompatible polymers [16]. Additionally, this approach represents a green alternative to chemical modification methods, eliminating residual toxicity and the need for complex surface post-treatment [19]. For example, the group of Xie et al. demonstrated that by using femtosecond laser treatment, periodic micro-grooves coupled with nanopore clusters on PEEK surfaces are created that lead to enhanced osteogenic differentiation of mouse bone marrow mesenchymal stem cells (mBMSCs), evidenced by increased cell viability and alkaline phosphatase activity—indicative of improved osseointegration potential [20]. Luo and coworkers, on the other hand, proved that a two-step surface activation method: femtosecond laser structuring followed by hydroxylation, generated microstructures, amorphous carbon, and -OH groups, enhancing hydrophilicity and surface energy that demonstrated superior ability to induce apatite deposition on PEEK—key for bone integration [21].

This work investigates the effect of femtosecond laser-induced micro- and nanoscale surface modification of PEEK, aiming to enhance the polymer’s hierarchical roughness, which could positively affect the bioactivity and osseointegration potential of the material. To achieve this, the development of various texturing designs of PEEK surfaces via femtosecond laser modification is performed. Through a systematic study of morphology, chemical composition, surface topography, and wettability (by means of SEM, EDX, XRD, microRaman, 3D profilometer, and WCA analyses), combined with SBF in vitro test, the goal is to develop personalized, functionally optimized PEEK surfaces, which, after profound in vitro testing as a further investigation could be implemented in bone tissue engineering. The results obtained from the morphological studies show that the created laser-induced surface designs of the bone PEEK implants can lead to improved surface properties, which could, as a next step, support the osteointegration capacity of the as-created micro/nano-structured PEEK scaffolds in the engineering of personalized bone tissue.

## 2. Materials and Methods

### 2.1. PEEK Substrate Preparation

Commercial medical polyetheretherketone (PEEK) natural rods (diameter 12 mm, Ensinger GmbH Sitz, Nufringen, Germany) were used as substrate material. Before laser processing, the rods were cut into tablet samples (thickness of 5 mm). All specimens were polished and ultrasonically cleaned in 70% ethanol (10 min) and deionized water (10 min), then dried in air before femtosecond laser surface functionalization was performed.

### 2.2. Femtosecond Laser Surface Modification

**Figure 1:**
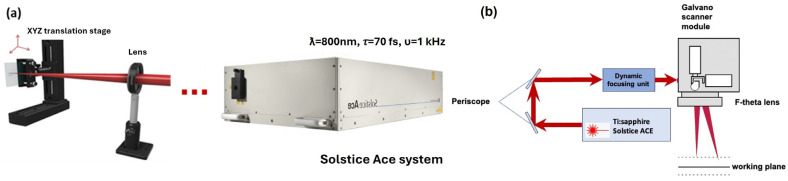
Schematic representation of the Solstice ACE femtosecond laser system integrated with (**a**) XYZ translation stage and (**b**) Scanlab Intelliscan scanning module.

Surface structuring was performed via a Ti:sapphire femtosecond laser system (Solstice ACE, MKS Spectra-Physics, Milpitas, CA, USA), operating at a central wavelength of ƛ = 800 nm, a pulse duration of *τ* = 70 fs, a repetition rate of υ = 1 kHz, and an output power of P = 6 W. The system is equipped with an XYZ motorized translation stage (Thorlabs, Newton, NJ, USA) for raster scanning of the samples, controlled via Kinesis v1.14.45 software (Thorlabs, Newton, NJ, USA). To improve processing speed and enable greater flexibility in design variation, the laser beam is guided through a SCANLABS galvanometric scanning head integrated with a Z-module and focused on the sample surface using a 160 mm f-theta lens, resulting in a spot diameter of d~25 µm. This configuration allows high-precision processing of complex 3D geometries, including models with irregular shapes—Figure 1.

After performing a parametric study by mounting the PEEK samples on the XYZ translation stage and raster scanning of the perpendicularly positioned laser beam on the sample surface performed in ambient air, the applied parameters of the femtosecond laser irradiation (frequency, power of the laser beam P = 20–80 mW, different focusing modes, hatch spacings dx = 45–100 µm, scanning velocities V = 3.44–32 mm/s and angles of incidence of the laser beam on the samples) were optimized to determine the conditions for achieving surface modifications with minimal thermal effects, preventing unwanted deformation or mechanical defects, and improving surface characteristics favorable for cell adhesion [14,15,17,18]. After optimizing the parameters of the applied femtosecond laser irradiation in a narrower range (P = 20–40 mW, V = 3.44–7.6 mm/s), different multilevel structuring designs were developed on the PEEK samples by means of the galvanometric scanning head. The processed samples were ultrasonically cleaned in 70% ethanol and dried in air to remove ablation debris. The ultrasonic cleaning of laser-processed PEEK samples in 70% ethanol was performed to remove ablation debris, as ethanol is known to effectively eliminate surface contaminants without inducing chemical alterations or degradation of PEEK [22].

### 2.3. Surface Morphology and Topography Characterization

The surface morphology of PEEK samples treated with femtosecond (fs) laser radiation was investigated using scanning electron microscopy (SEM) on a Hitachi SU8020 instrument (Hitachi High-Technologies Corporation, Tokyo, Japan). The device is equipped with a field-emission electron source and an SE (UL) detector. Before imaging, a thin carbon coating of around 20 nm was applied to the specimens by electron sputtering in a vacuum. SEM micrographs were then acquired at magnifications from 500× up to 5.0 k× for each type of surface modification. To evaluate surface roughness and topography, 3D measurements were performed with a Zeta-20 3D optical profiler (Zeta Instruments, KLA, Milpitas, CA, USA). This system functions as a fully integrated microscope (with magnifications ranging from 5× to 100×) and generates 3D surface maps by scanning across a defined vertical depth. The Zeta Optics module records precise XY positions and *Z*-axis heights for each scanning step, achieving a vertical resolution of less than 1 nm. These measurements produce detailed 3D composite images, from which roughness parameters are extracted: Ra (linear roughness, defined as the mean deviation of surface height from the central line according to DIN 4776) and Sa (areal roughness, extending Ra to the full surface area). For each sample, Sa values represent the average of five independent measurements, carried out on both laser-modified and untreated regions of PEEK specimens. For improved visualization and data analysis, the free software ProfilmOnline v2.0.0 was additionally employed (https://www.profilmonline.com) (accessed on 10 March 2025). 

### 2.4. Wettability Assessment

Wettability tests were carried out in an ambient air environment using a DSA100 Drop Shape Analyzer (KRÜSS GmbH, Heidelberg, Germany), a video-based optical contact angle measurement system. Dynamic water contact angle (WCA) measurements were conducted at room temperature by placing a 2 µL droplet of deionized water (dH_2_O) on the PEEK surface and monitoring its spreading for a period of 3 min. The contact angles were determined using the ADVANCE V.1.7.2.2 software (KRÜSS GmbH, Hamburg, Germany), which calculates values by fitting droplet shapes to the Young–Laplace equation. For each femtosecond (fs) laser-structured PEEK specimen, measurements were taken at three separate positions on five different samples; the results were then averaged and referred to those taken from the control PEEK surface. The standard deviation (SD) and Standard Error of the Mean WCA (SEM) were also calculated.

### 2.5. Structural Analysis and Chemical Evaluation

To determine the crystalline phase, degree of crystallinity (DOC), and the average size of crystal domains in the biopolymer matrices, X-ray diffraction (XRD) analysis was carried out over a 2θ range of 5–50° (step size of 0.013oθ2, in continuous scan mode and count time per step of 93.33 s). Measurements were performed using a Philips PW1050 diffractometer (Philips, Amsterdam, The Netherlands) equipped with a copper anode and a secondary monochromator on the diffracted beam. The resulting diffraction patterns were examined to assess potential changes in crystallinity resulting from femtosecond laser treatment. Phase identification was obtained via QualX2.0 software through the Crystallography Open Database (https://www.crystallography.net/). Additionally, microRaman spectroscopy was employed to characterize the samples. A LabRAM HR Visible spectrometer (HORIBA Jobin Yvon, Kyoto, Japan) with a He–Ne laser (633 nm) and an Olympus BX41 microscope was used to acquire Raman spectra, applying exposure times of 10–20 s and magnifications between 50× and 100×.

### 2.6. Osseointegration Potential Assessment

A Simulated Body Fluid (SBF) test was conducted as a first step in assessing both the bone-bonding capability and the potential of femtosecond (fs) laser-structured PEEK implants to promote cell growth and tissue regeneration by investigating apatite deposition on PEEK scaffolds. For this purpose, selected fs-treated polymer specimens along with untreated control samples were subjected to an in vitro SBF assay (five replicas each). The samples were incubated at 37 °C in freshly prepared SBF, following Kokubo’s protocol [23], for a period of two weeks. Each group of PEEK specimens was immersed in 1.5 mL of solution, and the formation of calcium phosphate on the surface was evaluated at three time points (days 3, 7, and 14) via SEM. The SBF medium was replenished every three days with a freshly prepared solution. Energy-dispersive X-ray spectroscopy (EDX) was used to analyze the growth of apatite crystals by evaluating the elemental distribution (area EDX maps taken at 0.5 k× magnification) and composition of the examined regions, expressed in weight (wt.%) percentages (area EDX spectra taken at 0.5 k× magnification).

## 3. Results

### 3.1. PEEK Surface Morphology and Roughness After Femtosecond Laser Structuring

**Figure 2:**
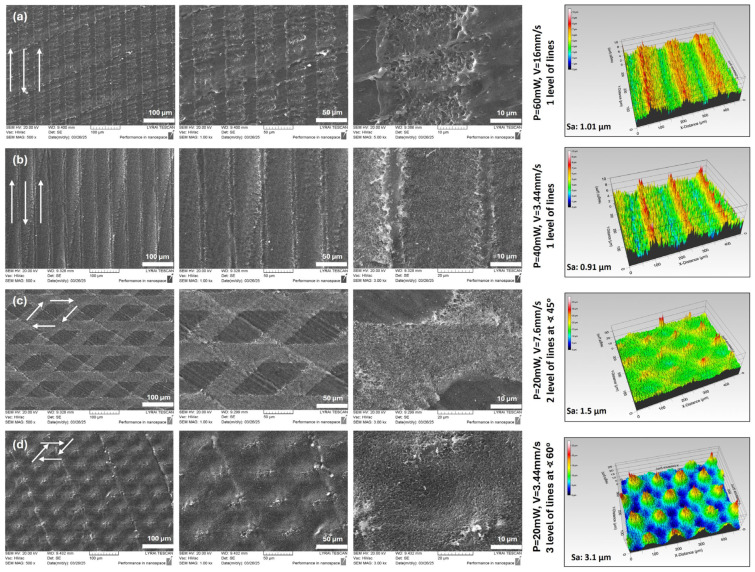
Representative SEM micrographs (500×, 1.0 and 5.0 k×) and surface area (Sa) roughness 3D real-color images of fs laser multilevel micro-structured designs of PEEK samples: (**a**) P = 60 mW, V = 16 mm/s, 1 level of lines; (**b**) P = 40 mW, V = 3.44 mm/s, 1 level of lines; (**c**) P = 20 mW, V = 7.6 mm/s, 2 levels of lines at ∢ 45°; (**d**) P = 20 mW, V = 3.44 mm/s, 3 levels of lines at ∢ 60°. The arrows indicate the direction of the laser scanning path.

**Figure 3:**
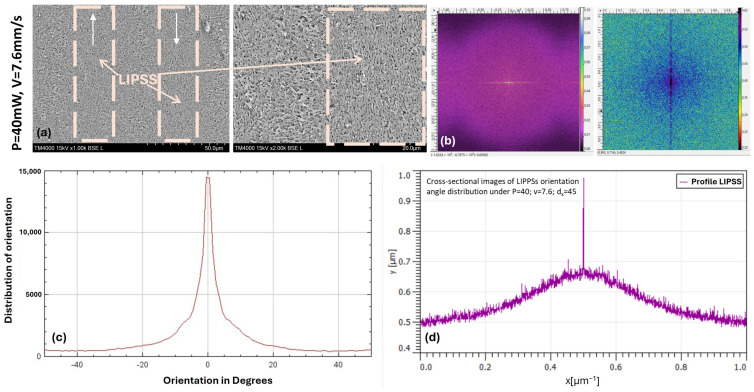
(**a**) SEM images (1.0 and 2.0 k×) of fs laser nano-structured designs of PEEK samples in the form of LIPSS; (**b**) Two-dimensional (2D) fast Fourier transformations (FFT) and (**c**) angular dispersion (θ) of LIPSS on PEEK; (**d**) Spatial period (Λ) of LIPSS obtained by 2D FFT analyses. The white arrows indicate the direction of the laser scanning path.

A series of laser powers (P = 20–80 mW), scanning velocities (V = 3.44–32 mm/s), and hatch spacings (dx = 45–100 µm) were applied to generate different raster and multilevel surface topographies. Figure 2 is a compilation of representative SEM images and 3D real-color profilometry maps of the obtained hierarchical designs. Femtosecond laser processing resulted in well-defined micro- and nanoscale surface architectures on the PEEK substrates. At lower laser powers and fast velocity (P = 40 mW, V = 7.6 mm/s), after raster scanning, the surface displayed parallel microgrooves with periodic nanoscale ripples, consistent with laser-induced periodic surface structures (LIPSS) formation—Figure 3a [13,24]. Increasing the power (P = 60 mW) produced deeper ablation features and hierarchical patterns composed of microcavities overlaid with nanopatterned ridges, resulting in rougher hierarchical structures. Higher the power, Figure 2a and lower the velocity applied, Figure 2b, the deeper and V-formed channels were obtained, as more material was ejected during ablation. Similar results were obtained when different topological designs were produced by overlapping several layers of laser-generated grooves at various angles—Figure 2c,d.

In addition to multilayered designs with tunable interlayer angles, a microstructured ‘Trulli-like landscape’ featuring distinct conical formations was also created—Figure 2d. Nanoscale morphology was observed both on the top and between the generated conical microstructures. The ultrashort pulse duration minimized thermal degradation, as no significant melting zones or carbonaceous debris were observed in SEM images. These morphological differences are attributed to the interplay between multiphoton absorption and localized photomechanical effects, allowing for the precise tuning of surface roughness by adjusting the laser power and scanning speed [25]. 3D optical profilometry confirmed that laser structuring significantly increased the average surface roughness (Sa) from the baseline polished PEEK value of 0.57 µm to values in the range 0.9–3.1 µm, depending on the laser processing parameters (Figure 2). The correlation between hatch spacing, velocity and roughness was notable: smaller hatch distances (dx = 45 µm) promoted overlapping ablation craters, leading to more uniform texture formation (Figure 3a), while larger hatch spacings (dx = 100 µm) preserved distinct groove morphology, which was an advantage when multilayered designs were created—Figure 2c,d—cell alignment and navigation could be easily monitored in vitro [26]. Higher velocities (16–32 mm/s), on the other hand, led to more noticeable differentiation of the singular ablation craters with respect to their smutter fusion and groove formation when lower laser velocities (3–7.6 mm/s) were applied. These results align with recent reports highlighting the importance of topographical cues in guiding osteoblast adhesion and differentiation [15,23]. In their elaborate review, Touya and coworkers examine the effect of nanotopography on cell–substrate interactions [26]. At the same time, the group of Legerstee explains in detail the mechanism of cellular adherence to surfaces via integrin-mediated focal contacts and the importance of the angle at which actin stress fibers promote stable adhesion to the substrate [27]. According to the performed literature survey, the optimal angle of the generated structures on the surface at which a stable contact with the biocompatible material is formed falls roughly within the range of 20–60°—Figure 2c,d [26,27].

To primarily analyze the obtained LIPSSs, the SEM images (Figure 3) were further explored using discrete two-dimensional fast Fourier transform analysis (2D-FFT), which was performed via open-source software (Gwyddion, Version 2.68)—Figure 3b. Low Spatial Frequency LIPSS (LSFL), perpendicular to the laser beam polarization with a period Λ LSFL larger than λ/2—approx. 500 nm were generated on PEEK polymer samples at P = 40 mW, v = 7.6 mm/s, dx = 45 µm—Figure 3d [28]. The average dispersion of the obtained LIPSSs’ orientation angle (DLOA) value is around 0: δθ~0°, which corresponds to the creation of nanostructures on the PEEK samples with very high regularity—Figure 3c [28]. Such high structural regularity is crucial for applications where uniform surface topography is desired, including tissue engineering scaffolds, cell alignment studies, or wetting behavior modulation [29]. The combination of low DLOA and a controlled LSFL period suggests that the applied laser parameters (P = 40 mW, v = 7.6 mm/s, dx = 45 µm) are within an optimal window for reproducible and precise nanostructuring of PEEK.

The findings presented demonstrate that careful tuning of laser power, scanning velocity, and hatch spacing can simultaneously produce ordered nanoscale surface textures on PEEK with minimal defects, which is advantageous for downstream biological applications [30]. The perpendicular orientation of LSFL relative to the polarization of the laser beam may further enable guided anisotropic interactions, such as cell alignment along surface patterns, thereby opening avenues for tailored surface-functionalized polymeric implants.

### 3.2. Wettability Evaluation

**Figure 4:**
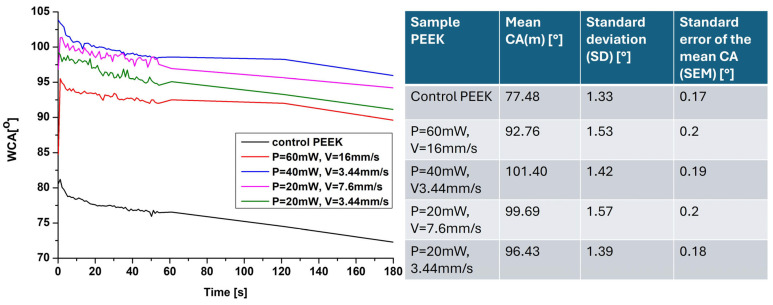
Dynamic WCA evaluation of the fs laser-created designs of PEEK samples for a period of 3 min. The mean value of the WCA [°], standard deviation (SD) [°], and standard error of the mean CA (SEM) [°] are also provided.

Water contact angle (WCA) measurements demonstrated a strong dependence on laser-induced roughness (Figure 4). Polished PEEK exhibited a moderately hydrophilic character (mean WCA = 77.5°). Laser treatment increased the mean contact angle value approximately by ~15°, indicating enhanced hydrophobicity. Notably, surfaces exhibiting hierarchical micro/nano structures demonstrated WCAs exceeding 105°, characteristic of very hydrophobic behavior. The presence of air pockets between the micro/nano structures, observed in SEM and 3D roughness images, can contribute to the hydrophobic behavior by trapping air and reducing the area of contact between the water droplet and the surface. This phenomenon is consistent with the Cassie-Baxter model, which describes the behavior of water droplets on rough surfaces with trapped air [11,30]. This causes the water droplet to initially spread unevenly within the laser-created microstructures, resulting in fluctuations in the WCA values and the irregular profile observed in the graph during the first 60 s of droplet application. The change in wettability can also be attributed to the possible introduction of oxygen-containing functional groups, resulting from laser-induced bond scission and subsequent atmospheric oxidation, as also observed in earlier femtosecond laser studies on polymers [5,11]. These chemical modifications can alter the surface energy, contributing to changes in wettability. Studies have shown that such surface transformations can lead to increased hydrophobicity, as the formation of functional groups like carbonyls and hydroxyls can reduce surface energy [31]. Such changes are crucial for applications where controlled wettability is desired, as are those in tissue engineering scaffolds, cell alignment studies, and modulation of wetting behavior [32,33].

### 3.3. Structural and Chemical Analysis (XRD, microRaman)

**Figure 5:**
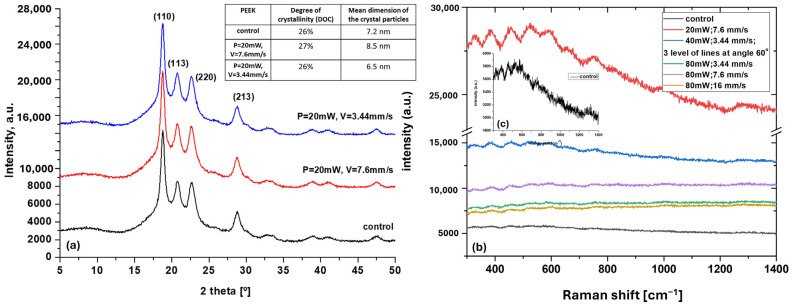
Representative (**a**) XRD and (**b**) micro-Raman spectra of the fs laser-structured PEEK with respect to control samples—(**c**) scale up of the control PEEK micro-Raman spectrum at lower intensity (a.u.).

The results obtained from the XRD analysis performed on selected fs laser-structured PEEK samples compared to control ones are given in Figure 5a. PEEK is a semicrystalline thermoplastic biopolymer with the phase of orthorhombic crystalline form [34]. XRD patterns confirmed no major crystalline phase changes after laser processing, as all characteristic diffraction peaks at 2θ = 19.1°, 21°, and 23.1° corresponding to the (110), (113), and (220) planes remained unchanged. The peak around the diffraction angle 2θ of 29°, assigned to the (213) plane of PEEK, also did not change its position or intensity under the influence of fs laser radiation [35]. The degree of crystallinity (DOC), also presented in the figure (table), and the average size of crystal domains remain almost unchanged after laser ablation, indicating negligible surface amorphization restricted to the ablated regions. This observation aligns with the non-thermal nature of ultrashort pulse interactions, where material removal is primarily driven by photomechanical effects and multiphoton ionization [25].

The micro-Raman spectra, also presented on the same figure (b), complement the XRD data, stating once again that fs laser radiation does not cause a major change in the chemical composition of the PEEK polymer, as no significant distribution of the peaks’ position is observed after fs laser ablation in comparison to the control sample. As Numata et al. explain in their elaborate Raman study on polyetheretherketone [36], the peaks in the mid-frequency region of the PEEK spectrum have been previously studied in detail and are discussed elsewhere [37]. Nevertheless, a noticeable change in the intensity of the characteristic peaks is monitored after laser radiation is applied (a scale-up of the control PEEK micro-Raman spectrum at lower intensity is presented in Figure 5c for better visualization), which could be attributed to the change in surface roughness and topography (micro/nano-structures affect the surface scattering and optical absorption, modifying peak intensities), rather to alternation in chemical composition of the PEEK polymer [38]. Moreover, fs laser radiation could lead to partial biopolymer chain alignment, affecting vibrational mode intensities, due to the localized heating and rapid cooling nature of the ultra-fast fs interaction with the material [39].

### 3.4. SBF in Vitro Test

**Figure 6:**
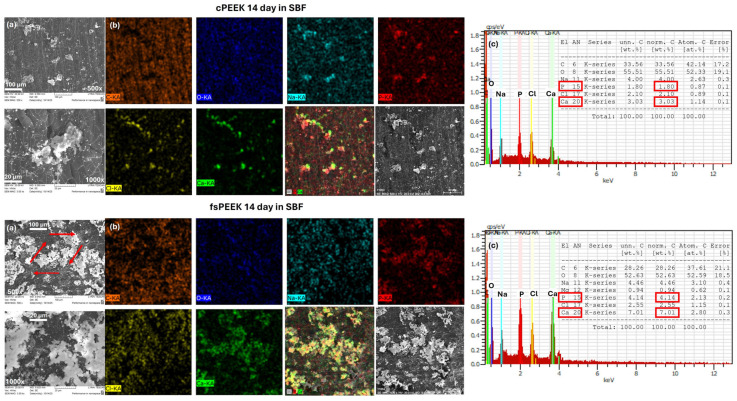
SBF in vitro test—(**a**) SEM images at 0.5 and 1.0 k×, (**b**) EDX maps (at 0.5 k×), and (**c**) EDX spectra (at 0.5 k×) of control cPEEK (upper figure panel) and laser-structured fsPEEK (lower figure panel) after incubation in SBF for 14 days. The red arrows indicate the direction of the laser scanning path.

To assess the potential of PEEK to support cell growth and tissue regeneration and to examine the potential of fs surface treatments designed to improve implant integration, a simulated body fluid test was performed as a first step for biological evaluation (Figure 6). On the SEM micrographs of control cPEEK and laser-structured fsPEEK (Figure 6a), the microapatite crystals are easily visible on the surface of the samples, in vitro incubated in Simulated Body Fluid for a period of two weeks. Nevertheless, the quantity of the deposited calcium phosphate is much higher on the laser-treated PEEK. The SEM results are complemented by those obtained via the simultaneously performed EDX mapping, demonstrating not only the higher quantity but also the homogeneous distribution of Ca and P elements on the surface of the laser-treated PEEK samples, compared to the control ones—Figure 6b. The results suggest that femtosecond laser structuring produces surface topographies that significantly enhance apatite formation. These findings are consistent with previous reports and could be attributed to the presence of hierarchical surface features that serve as preferential sites for calcium phosphate nucleation and growth, that could have a positive effect on osteoconductivity and a stronger bone–implant interfacial bond [40]. In their work, Oyane and colleagues [41] reported that laser-assisted wet coating techniques applied to PEEK substrates promoted the formation of a dense hydroxyapatite layer when immersed in SBF. The authors attributed this to laser-induced surface roughening, which supports heterogeneous nucleation of calcium phosphate and enhances subsequent interactions with osteoblastic MC3T3-E1 cells, thereby improving the material’s bioactivity and cellular compatibility.

As can be seen from the EDX spectra in Figure 6c, Ca and P are detected in both samples, but the concentration [wt.%] of the elements is much higher on the laser-structured PEEK surface—[P]-4.14 wt.% and [Ca]-7.01 wt.%, compared to [P]-1.8 wt.% and [Ca]-3.03 wt.% for cPEEK. Furthermore, the monitored Ca percentage is approximately twice that of P concentration, a ratio that aligns closely with the stoichiometric composition of calcium phosphate phases typically found in bone tissue [40].

For better comprehension and enhanced clarity of the results obtained in the current study, a comparison of the surface roughness (Sa), wettability (mean WCA), change in the degree of crystallinity and elemental composition (with respect to Ca/P ratios after SBF test) of representative fs-laser-treated PEEK surfaces in relation to control samples is presented in Table 1.

## 4. Discussion

As can be seen from the performed analysis and the summarized results in Table 1, the present study demonstrates that femtosecond laser processing can produce reproducible micro/nanostructured PEEK surfaces with tunable morphology, roughness and conserved chemical structure. The absence of significant chemical or crystallographic alteration (Figure 5a,b) highlights the non-thermal nature of femtosecond ablation, consistent with previous studies reporting that ultrashort pulses minimize polymer degradation and carbonization [25,34,38]. On the other hand, the high degree of tunability of WCA values is very important for biomedical applications [32,33]. Recent investigations support these findings, emphasizing the importance of ultrafast-laser-induced hierarchical structuring in modulating polymer surface properties for biomedical applications [42,43].

The observed increase in roughness and hydrophobicity (Section 3.2) aligns with current literature describing that combined micro/nano topographies promote hydrophobic or amphiphilic behavior, depending on the oxidation degree and local chemistry [44]. These topographic and chemical cues are key for controlling protein adsorption and subsequent cell adhesion mechanisms [26,27]. A similar trend has been reported by Luo et al., who demonstrated that hierarchical roughness on PEEK scaffolds improved early osteoblast adhesion and mineralization by promoting enhanced fibronectin adsorption and osteoblastic cell spreading [21].

Beyond hydrophobicity, surface energy modulation induced by fs-laser treatment may directly influence the adsorption of serum proteins such as fibronectin and vitronectin, which in turn regulate integrin-mediated cell attachment. In their elaborate review, the group of Cai and coworkers demonstrated that controlled nanoscale patterning on polymeric implants could adjust focal adhesion maturation and intracellular signaling, improving both cytoskeletal organization and matrix deposition [45]. These findings correlate well with the high regularity of the LIPSS obtained in the present study, where orientation and periodicity are likely to provide consistent anchoring sites for adhesion complexes.

In terms of wettability, the Cassie–Baxter wetting regime observed here suggests that trapped air pockets contribute to hydrophobicity. However, post-laser oxidation may introduce functional oxygen groups, gradually shifting the surface toward hydrophilicity during physiological exposure. Such temporal wetting transitions have been confirmed in recent works examining the dynamic surface aging of fs-treated polymers [46,47]. The presence of polar oxygen functionalities can increase the polar component of surface energy, which may further enhance biomolecule adsorption and support cell proliferation under wet biological conditions. Comparable behavior has been documented by Kryszak et al., who showed that femtosecond-laser-treated poly(L-lactide) surfaces exhibited tailored wettability and enhanced osteoblast adhesion and cell viability [48]. In the context of the present study, future work will focus on systematically examining the potential wettability evolution of fs laser-structured PEEK and its correlation with protein adsorption kinetics and cell response.

The XRD and Raman analyses confirmed structural integrity, indicating that photomechanical ablation predominated. These findings corroborate the growing understanding that femtosecond laser structuring allows for precise morphological control without compromising polymer crystallinity—a crucial requirement for maintaining mechanical strength in load-bearing implants [49]. Recent femtosecond-laser microstructuring studies on semi-crystalline PEEK polymers have demonstrated that ablation can be performed with minimal thermal damage and with preservation of crystalline peaks, suggesting that polymer crystallinity and thus mechanical integrity can be maintained. Although explicit studies on fatigue resistance and modulus retention remain limited, crystallinity preservation is a promising indicator of load-bearing performance [8,21,50].

The enhanced apatite formation seen after SBF incubation is consistent with the literature linking increased surface roughness, hierarchical structuring, and oxygen incorporation with improved nucleation of calcium phosphates [40,41]. Recent reports show that micro and nanoscale periodicity can modulate osteogenic differentiation pathways through focal adhesion and mechanotransduction [51]. For example, Gao et al. demonstrated that hierarchical topographies on polymer–based matrices accelerated mineralization and enhanced osteogenic differentiation in osteoblasts [52]. Moreover, Luo et al. proved that femtosecond-laser-etched PEEK surfaces exhibited enhanced apatite deposition and improved cell proliferation, attributed to the synergistic effect of micro/nano topography and surface chemistry [8].

It is now understood that surface topography, chemistry, and wettability act synergistically to influence protein adsorption, ion exchange, and subsequent cell–material interactions [53]. The hierarchical conical and grooved designs produced in the current research (Figure 2) provide multiple length scales of surface roughness that may have a positive effect on initial protein attachment and later osteogenic differentiation. Such multiscale effects have been described in depth in the works of Böker et al. [54] and Bressel et al. [55], who reported that femtosecond laser microcones on titanium enhanced early osteogenic signaling, even in the absence of biochemical coatings. Comparable findings on polymeric systems confirm that similar physical cues could drive comparable biological responses when nanoscale order and chemistry are properly balanced [21,44,45].

Furthermore, as an additional step, laser-induced modifications could be coupled with post-processing strategies such as plasma oxidation, peptide immobilization, or hydrothermal crystallization to further enhance PEEK’s osteoinductive performance. For instance, Dondani et al. demonstrated that plasma treatment improves surface energy and apatite formation, suggesting a potential synergistic effect when both approaches are combined [56]. The potential to combine topographical and chemical activation suggests an emerging platform approach for customizing PEEK-based biomaterials. Overall, these results reinforce the concept that femtosecond laser surface processing offers a tunable, non-chemical route to optimize PEEK surface properties, which, after proper in vitro and in vivo testing, could find application in orthopedic and dental implantology.

Building on these results, the research group will next undertake systematic biological validation of the femtosecond-laser-modified PEEK surfaces, beginning with in vitro cellular experiments and extending to in vivo studies in future work. Planned in vitro assays will include quantitative evaluation of osteoblast adhesion, proliferation, orientation and differentiation. Cytotoxicity, ALP (Alkaline Phosphatase) activity and gene expression profiling of osteogenic markers, providing insight into the molecular mechanisms governing cell–material interactions, are also planned to be performed. The combination of micro/nano hierarchical topographies, stable structural integrity, and improved HA deposition capacity positions fs-laser-modified PEEK as a promising candidate for next-generation biomaterials engineered for osteointegration and long-term biocompatibility. Moreover, considering the rapid advances in high-precision laser scanning and in situ feedback control, the scalability and reproducibility of fs-textured PEEK surfaces could soon enable clinical-grade manufacturing of implants, aligning with current trends toward patient-specific regenerative solutions [57].

## 5. Conclusions

This study demonstrates that femtosecond laser micro/nanostructuring is a highly effective approach for developing surface features potentially favorable for the bioactivity of PEEK bone-resembling surfaces. By precisely controlling laser parameters, a wide range of hierarchical surface morphologies was generated, providing fine adjustment of roughness and wettability without altering the bulk properties of the material. Importantly, the structural integrity and crystallinity of PEEK remained largely unaffected, confirming the non-thermal, localized nature of femtosecond laser processing. These results highlight the future potential of femtosecond laser technology as a reproducible, environmentally friendly, and scalable method for surface processing of polyetheretherketone-based implants. The structures created on the PEEK scaffolds led to increased apatite deposition that may promote conditions favorable for osteoblast adhesion and viability. Such tailored surface modifications can contribute to the future development of next-generation orthopedic and spinal substitutes with enhanced osseointegration capacity, advancing bone tissue engineering solutions.

## Figures and Tables

**Table 1:** Comparative data of Sa, WCA, DOC, and Ca/P ratios after SBF test of control and laser-structured PEEK tablets.

PEEK	Sa [µm]	Mean WCA [°]	DOC [%]	Ca/P After SBF
control	0.57	77.48	26	1.68
P = 20 mW, v = 7.6 mm/s	1.5	99.96	27	1.69
P = 20 mW, v = 3.44 mm/s	3.1	96.43	26	1.68
LIPSS	0.97	102	26	1.67

## Data Availability

The original contributions presented in the study are included in the article; further inquiries can be directed to the corresponding authors.

## Abbreviations

The following abbreviations are used in this manuscript:

LIPSS	laser–induced periodic surface structures
PEEK	polyetheretherketone
fs	femtosecond
SS	stainless steel
SEM	scanning electron microscopy
EDX	energy-dispersive X-ray spectroscopy
XRD	X-ray Diffraction
DOC	degree of crystallinity
WCA	water contact angle
mBMCs	mouse bone marrow mesenchymal stem cells
SBF	Simulated Body Fluid
2D-FFT	two-dimensional fast Fourier transform analysis
LSFL	Low Spatial Frequency LIPSS
DLOA	dispersion of LIPSSs’ orientation angle
SD	Standard deviation

## References

[1] Kurtz S.M., Devine J.N. (2007). PEEK biomaterials in trauma, orthopedic, and spinal implants. Biomaterials.

[2] Ma R., Tang T. (2014). Current strategies to improve the bioactivity of PEEK. Int. J. Mol. Sci..

[3] Yin W., Chen M., Bai J., Xu Y., Wang M., Geng D., Pan G. (2022). Recent Advances in Orthopedic Polyetheretherketone Biomaterials: Material Fabrication and Biofunction Establishment. Smart Mater. Med..

[4] Bekmurzayeva A., Duncanson w.j., Azevedo H.S., Kanayeva D. (2018). Surface modification of stainless steel for biomedical applications: Revisiting a century-old material. Mater. Sci. Eng. C.

[5] Sun H., Li J., Liu M., Yang D., Li F. (2022). A Review of Effects of Femtosecond Laser Parameters on Metal Surface Properties. Coaings.

[6] Harting R., Barth M., Bührke T., Pfefferle R.S., Petersen S. (2017). Functionalization of Polyetheretherketone for Application in Dentistry and Orthopedics. BioNanoMaterials.

[7] Cheng B.C., Koduri S., Wing C.A., Woolery N., Cook D.J., Spiro R.C. (2018). Porous Titanium-Coated Polyetheretherketone Implants Exhibit an Improved Bone–Implant Interface: An In Vitro and In Vivo Biochemical, Biomechanical, and Histological Study. Med. Devices (Auckl.).

[8] Luo F., Mao R., Huang Y., Wang L., Lai Y., Zhu X., Fan Y., Wang K., Zhang X. (2022). Femtosecond Laser Optimization of PEEK: Efficient Bioactivity Achieved by Synergistic Surface Chemistry and Structures. J. Mater. Chem. B.

[9] Xie D., Xu C., Ye C., Mei S., Wang L., Zhu Q., Chen Q., Zhao Q., Xu Z., Wei J. (2021). Fabrication of Submicro-Nano Structures on Polyetheretherketone Surface by Femtosecond Laser for Exciting Cellular Responses of MC3T3-E1 Cells/Gingival Epithelial Cells. Int. J. Nanomed..

[10] Riveiro A., Maçon A.L.B., del Val J., Comesaña R., Pou J. (2018). Laser Surface Texturing of Polymers for Biomedical Applications. Front. Phys..

[11] Obilor A.F., Pacella M., Wilson A., Silberschmidt V.V. (2022). Micro-texturing of polymer surfaces using lasers: A review. Int. J. Adv. Manuf. Technol..

[12] Graham C.L.B., Newman H., Gillett F.N., Smart K., Briggs N., Banzhaf M., Roper D.I. (2021). A Dynamic Network of Proteins Facilitate Cell Envelope Biogenesis in Gram-Negative Bacteria. Int. J. Mol. Sci..

[13] Bonse J., Höhm S., Kirner S.V., Rosenfeld A., Krüger J. (2017). Laser-Induced Periodic Surface Structures— A Scientific Evergreen. IEEE J. Sel. Top. Quantum Electron..

[14] Sugioka K., Cheng Y. (2014). Ultrafast lasers—Reliable tools for advanced materials processing. Light Sci. Appl..

[15] Anselme K., Davidson P., Popa A.M., Giazzon M., Liley M., Ploux L. (2010). The interaction of cells and bacteria with surfaces structured at the nanometre scale. Acta Biomater..

[16] Villapún V.M., Man K., Carter L., Penchev P., Dimov S., Cox S. (2023). Laser texturing of additively manufactured implants: A tool to programme biological response. Biomater. Adv..

[17] Fu Q., Gabriel M., Schmidt F., Müller W.-D., Schwitalla A.D. (2021). The impact of different low-pressure plasma types on the physical, chemical and biological surface properties of PEEK. Dent. Mater..

[18] Lee J.H., Park Y.J., Lim Y.J., Huh J.B. (2021). Effect of Acid-Etching Duration on the Adhesive Performance of Printed Polyetheretherketone to Veneering Resin. Polymers.

[19] Hou C., An J., Zhao D., Ma X., Zhang W., Zhao W., Wu M., Zhang Z., Yuan F. (2022). Surface Modification Techniques to Produce Micro/Nano-scale Topographies on Ti-Based Implant Surfaces for Improved Osseointegration. Front. Bioeng. Biotechnol..

[20] Xie H., Zhang C., Wang R., Tang H., Mu M., Li H., Guo Y., Yang L., Tang K. (2021). Femtosecond laser-induced periodic grooves and nanopore clusters make a synergistic effect on osteogenic differentiation. Colloids Surf. B Biointerfaces.

[21] Luo F., Li D., Huang Y., Mao R., Wang L., Lu J., Ge X., Fan Y., Zhang X., Chen Y. (2023). Efficient osteogenic activity of PEEK surfaces achieved by femtosecond laser–hydroxylation. ACS Appl. Mater. Interfaces.

[22] Pidhatika B., Widyaya V.T., Nalam P.C., Swasono Y.A., Ardhani R. (2022). Surface Modifications of High-Performance Polymer Polyetheretherketone (PEEK) to Improve Its Biological Performance in Dentistry. Polymers.

[23] Kokubo T., Takadama H. (2006). How useful is SBF in predicting in vivo bone bioactivity?. Biomaterials.

[24] Terakawa M. (2018). Femtosecond Laser Processing of Biodegradable Polymers. Appl. Sci..

[25] Shin S. (2024). Review of high-precision femtosecond laser materials processing for fabricating microstructures: Effects of laser parameters on processing quality, ablation efficiency, and microhole shape. J. Laser Appl..

[26] Touya N., Al-Bourgol S., Désigaux T., Kérourédan O., Gemini L., Kling R., Devillard R. (2023). Bone laser patterning to decipher cell organization. Bioengineering.

[27] Legerstee K., Houtsmuller A.B. (2021). A layered view on focal adhesions. Biology.

[28] Sikora A., Faucon M., Gemini L., Kling R., Mincuzzi G. (2022). LIPSS and DLIP: From hierarchical to mutually interacting, homogeneous structuring. Appl. Surf. Sci..

[29] Bonse J., Gräf S. (2021). Ten open questions about laser-induced periodic surface structures. Nanomaterials.

[30] Cassie A.B.D., Baxter S. (1944). Wettability of porous surfaces. Trans. Faraday Soc..

[31] Chen Z., Zhou J., Cen W., Yan Y., Guo W. (2025). Femtosecond Laser Fabrication of Wettability-Functional Surfaces: A Review of Materials, Structures, Processing, and Applications. Nanomaterials.

[32] Cao S., Yuan Q. (2022). An Update of Nanotopographical Surfaces in Modulating Stem Cell Fate: A Narrative Review. Biomat. Transl..

[33] Han F., Meng Q., Xie E., Li K., Hu J., Chen Q., Li J., Han F. (2023). Engineered Biomimetic Micro/Nano-Materials for Tissue Regeneration. Front. Bioeng. Biotechnol..

[34] Yu S., Hariram K.P., Kumar R., Cheang P., Aik K.K. (2005). In vitro apatite formation and its growth kinetics on hydroxyapatite/polyetheretherketone biocomposites. Biomaterials.

[35] Song J., Liao Z., Shi H., Xiang D., Liu Y., Liu W., Peng Z. (2017). Fretting wear study of PEEK-based composites for bio-implant application. Tribol. Lett..

[36] Numata T., Ishikawa N., Shimada T., Gordon K.C., Yamaguchi M. (2024). Low-frequency Raman spectroscopy on amorphous poly(ether ether ketone) (PEEK). Materials.

[37] Doumeng M., Makhlouf L., Berthet F., Marsan O., Delbé K., Denape J., Chabert F. (2021). A comparative study of the crystallinity of polyetheretherketone by using density, DSC, XRD, and Raman spectroscopy techniques. Polym. Test..

[38] Numata T., Ishikawa N., Shimada T., Gordon K.C., Yamaguchi M. (2024). Evaluation of Spatial Distribution of Crystallinity Induced by Local Heating Using Low-Frequency Raman Spectroscopy on Polyether Ether Ketone (PEEK). Spectrosc. J..

[39] Hassan N.M., Migler K.B., Hight Walker A.R., Kotula A.P., Seppala J.E. (2021). Comparing polarized Raman spectroscopy and birefringence as probes of molecular scale alignment in 3D printed thermoplastics. MRS Commun..

[40] Dorozhkin S.V. (2009). Calcium orthophosphates in nature, biology and medicine. Materials.

[41] Oyane A., Nakamura M., Sakamaki I., Shimizu Y., Miyata S., Miyaji H. (2018). Laser-assisted wet coating of calcium phosphate for surface functionalization of PEEK. PLoS ONE.

[42] Stratakis E., Bonse J., Heitz J., Siegel J., Tsibidis G.D., Skoulas E., Papadopoulos G.A., Mimidis A., Joel A.-C., Comanns P. (2020). Laser engineering of biomimetic surfaces. Mater. Sci. Eng. R..

[43] Correa D.S., Almeida J.M.P., Almeida G.F.B., Cardoso M.R., De Boni L., Mendonça C.R. (2017). Ultrafast Laser Pulses for Structuring Materials at Micro/Nano Scale: From Waveguides to Superhydrophobic Surfaces. Photonics.

[44] Khoury J., Maxwell M., Cherian R.E., Bachand J., Kurz A.C., Walsh M., Assad M., Svrluga R.C. (2017). Enhanced bioactivity and osseointegration of PEEK with accelerated neutral atom beam technology. J. Biomed. Mater. Res. Part B.

[45] Cai S., Wu C., Yang W., Liang W., Yu H., Liu L. (2020). Recent advance in surface modification for regulating cell adhesion and behaviors. Nanotechnol. Rev..

[46] Vesel A., Zaplotnik R., Primc G., Mozetič M. (2020). Evolution of the Surface Wettability of PET Polymer upon Treatment with an Atmospheric-Pressure Plasma Jet. Polymers.

[47] Riveiro A., Soto R., Comesaña R., Boutinguiza M., del Val J., Quintero F., Lusquiños F., Pou J. (2012). Laser surface modification of PEEK. Appl. Surf. Sci..

[48] Kryszak B., Szustakiewicz K., Dzienny P., Junka A., Paleczny J., Szymczyk-Ziółkowska P., Hoppe V., Antończak A. (2022). Functionalization of the PLLA surface with a femtosecond laser: Tailored substrate properties for cellular response. Polym. Test..

[49] Daskalova A., Filipov E., Angelova L., Stefanov R., Tatchev D., Avdeev G., Sotelo L., Christiansen S., Sarau G., Leuchs G. (2021). Ultra-Short Laser Surface Properties Optimization of Biocompatibility Characteristics of 3D Poly-ε-Caprolactone and Hydroxyapatite Composite Scaffolds. Materials.

[50] Li Q., Perrie W., Potter R., Allegre O., Li Z., Tang Y., Zhu G., Dun L., Chalker P., Ho J. (2020). Femtosecond laser micro-structuring of amorphous polyether(ether)ketone at 775 nm and 387 nm. J. Phys. D Appl. Phys..

[51] Teo B.K.K., Wong S.T., Lim C.K., Kung T.Y.S., Yap C.H., Ramagopal Y., Romer L.H., Yim E.K.F. (2013). Nanotopography Modulates Mechanotransduction of Stem Cells and Induces Differentiation through Focal Adhesion Kinase. ACS Nano.

[52] Gao Q., Hou Y., Li Z., Hu J., Huo D., Zheng H., Zhang J., Yao X., Gao R., Wu X. (2021). mTORC2 Regulates Hierarchical Micro/Nano Topography-Induced Osteogenic Differentiation via Promoting Cell Adhesion and Cytoskeletal Polymerization. J. Cell. Mol. Med..

[53] Li D., Zheng Q., Wang Y., Chen H. (2014). Combining surface topography with polymer chemistry: Exploring new interfacial biological phenomena. Polym. Chem..

[54] Böker K.O., Kleinwort F., Klein-Wiele J.-H., Simon P., Jäckle K., Taheri S., Lehmann W., Schilling A.F. (2020). Laser Ablated Periodic Nanostructures on Titanium and Steel Implants Influence Adhesion and Osteogenic Differentiation of Mesenchymal Stem Cells. Materials.

[55] Bressel T.A.B., de Queiroz J.D.F., Gomes Moreira S.M., da Fonseca J.T., Filho E.A., Guastaldi A.C., Batistuzzo de Medeiros S.R. (2017). Laser-modified titanium surfaces enhance osteogenic differentiation of human mesenchymal stem cells. Stem Cell Res. Ther..

[56] Dondani J.R., Iyer J., Tran S.D. (2023). Surface Treatments of PEEK for Osseointegration to Bone. Biomolecules.

[57] Rendas P., Figueiredo L., Machado C., Mourão A., Vidal C., Soares B. (2023). Mechanical performance and bioactivation of 3D-printed PEEK for high-performance implant manufacture: A review. Prog. Biomater..

